# The CDC Colorectal Cancer Control Program, 2009–2015

**DOI:** 10.5888/pcd16.190336

**Published:** 2019-12-05

**Authors:** Djenaba A. Joseph, Amy DeGroff

**Affiliations:** 1 Centers for Disease Control and Prevention, National Center for Chronic Disease Prevention and Health Promotion, Division of Cancer Prevention and Control, Atlanta, Georgia

Colorectal cancer (CRC) is the second leading cause of cancer deaths in the United States among cancers that affect both men and women (1). In 2016, the most recent year for which data are available, more than 141,000 new cases of CRC were reported, and more than 52,000 people died of the disease (1). The public health impact of CRC due to years of potential life lost, the economic burden of lost productivity, and the costs associated with illness and treatment are substantial. In 2015, an estimated 766,000 person-years of life lost and $9.4 billion in lost earnings were attributed to CRC deaths, second only to lung cancer (2). Strong evidence indicates that screening can decrease CRC incidence and mortality by identifying and removing precancerous polyps and by detecting CRC early when treatment is more effective (3). If CRC is detected early, the 5-year survival rate (90%) is much higher than when it is detected late (14%) (1).

The US Preventive Services Task Force (USPSTF) recommends CRC screening for average-risk people aged 50 to 75 by fecal occult blood test (FOBT), fecal immunochemical test (FIT), a combination stool DNA and FIT test (FIT–DNA), computed tomographic colonography (CTC, or virtual colonoscopy), flexible sigmoidoscopy, or colonoscopy (3). Despite strong evidence for its effectiveness, too few eligible adults are screened for CRC. In 2016, 67% of adults aged 50 to 75 reported that they were up-to-date with CRC screening, whereas 26%, or approximately 22 million adults, reported that they had never been screened (4). Screening rates are lower among people who have a low annual household income, have no health insurance, have no regular health care provider, identify as a racial or ethnic minority, or have low levels of educational attainment (5).

The high public health burden of CRC indicates a need for population-level interventions to improve its prevention and control (2). Although large health systems have implemented programs and initiatives to improve the quality of CRC screening and treatment in their populations, coordinated, population-level public health efforts that reach most, or all, of the US population to address the burden of CRC have been limited (6). Examples of national or multistate efforts to increase CRC screening include programs or campaigns implemented by organizations such as the Centers for Disease Control and Prevention (CDC) and the American Cancer Society (ACS). CDC’s Screen for Life: National Colorectal Cancer Action Campaign is a national mass media and small media campaign that informs adults about the importance of getting screened for CRC (7). In 2014, the National Colorectal Cancer Roundtable, an organization founded by CDC and ACS to bring organizations together to coordinate efforts to address the burden of CRC, launched the 80% by 2018 campaign, which asked organizations of all types to pledge resources toward interventions to increase CRC screening rates (8). More than 1,500 organizations signed the pledge to participate (9). From 2013 through 2016, ACS implemented the Community Health Advocates Implementing Nationwide Grants for Empowerment and Equity (CHANGE) program, which funded primary care systems, faith-based organizations, and community-based organizations that partnered with federally qualified health centers to implement evidence-based interventions to increase breast and CRC screening with technical assistance from ACS field staff members (10). Finally, CDC’s National Comprehensive Cancer Control Program supports the development and implementation of cancer control plans, and partners with state, tribal, and territorial cancer coalitions to leverage resources to address cancer prevention and control, including efforts to increase use of CRC screening tests (11). Literature describing program design, implementation, or evaluation of these efforts is limited, suggesting the need for additional information about best practices to design, implement, and evaluate national or multistate efforts to increase CRC screening (6,8,10,11).

A collection of 5 articles published in 2019 in *Preventing Chronic Disease* describes the evaluation of CDC’s 2009–2015 Colorectal Cancer Control Program (CRCCP), including its implementation, outcomes, and costs. These articles contribute to the limited body of peer-reviewed literature about programmatic design approaches and best practices for large, multistate, population-level public health interventions to increase use of CRC screening tests.

## Program Overview

In 2004, CDC funded 5 sites to implement the Colorectal Cancer Screening Demonstration Program to assess the feasibility of public health approaches to address the burden of CRC and the low uptake of CRC screening tests among populations that traditionally have had limited access to health care services (12). This demonstration program was modeled after the long-standing National Breast and Cervical Cancer Early Detection Program (NBCCEDP), authorized by Congress to provide breast and cervical cancer screening and diagnostic services to low-income, uninsured, and underinsured women. The NBCCEDP demonstrated success in working with provider networks, community partners, professional organizations, and other partners to provide access to high-quality cancer screening and diagnostic services. On the basis of the success of the Colorectal Cancer Screening Demonstration Program and lessons learned from both the demonstration program and the NBCCEDP, in 2009 CDC launched the 5-year CRCCP to provide CRC screening tests to low-income, uninsured, and underinsured populations and to promote the importance of screening with the ambitious goal of increasing screening rates to 80%.

Through a competitive application process, CDC funded 22 states and 4 tribal organizations to implement the CRCCP. In July 2010, CDC funded an additional 3 states, bringing the total number of grantees to 29 (13). The CRCCP comprised 2 program components: 1) screening provision, which provided CRC screening tests for people with low incomes and no or limited health insurance, and 2) screening promotion, which involved activities to increase awareness and uptake of CRC screening on a population level.

For the screening provision component, grantees used a portion of their awards to fund clinical screening services. Grantees established contracts with health care providers to deliver screening to the priority population: asymptomatic people aged 50 to 64 who had an annual household income less than or equal to 250% of the federal poverty level and were uninsured or underinsured for CRC screening services. “Underinsured” was defined in various ways across grantees, but in general it referred to people who did not have insurance coverage for preventive services (eg, they had catastrophic health care coverage only) or could not afford copays or deductibles. People aged 65 or older were excluded from receiving these screening services because they were covered by Medicare. Grantees had the option to fund any CRC screening test indicated in the 2008 USPSTF recommendations (FOBT, FIT, flexible sigmoidoscopy, or colonoscopy). Additional program activities to support screening included patient outreach and awareness, patient navigation, provider education, quality assurance, and data management.

For the screening promotion component, grantees implemented evidence-based interventions (EBIs) identified in *The Community Guide* (14) to increase population-level use of CRC screening. At the time of program initiation, EBIs included client and provider reminders, provider assessment and feedback, reduction of structural barriers, and small media (15).

## Evaluation Design

CDC undertook an evaluation of the CRCCP to assess implementation, outcomes, and costs. Grantees also conducted local evaluations. CDC designed its evaluation on the basis of CDC’s Framework for Program Evaluation (16) and identified these goals:

- Describe how CRCCP grantees implement the program.
- Assess changes in key outcomes, including population-level CRC screening prevalence.
- Describe the costs of implementing the CRCCP for both screening provision and screening promotion.

Three unique data collection methods were used. To evaluate screening provision, a patient-level data set was developed (CRC clinical data elements, or CCDEs). To assess implementation of EBIs, CDC conducted an annual grantee survey, and to assess cost, grantees completed a cost assessment tool (Table).

**Table:** Evaluation Questions and Data Collection for CDC’s 2009–2015 Colorectal Cancer Control Program (CRCCP)

Evaluation Question	Data Collection Tool	Reporting Frequency	Unit of Measurement	Constructs or Variables
Is complete and timely screening delivered, and what are the screening outcomes?	Colorectal cancer clinical data elements (CCDEs)	Semi-annually	Patient	• Patient demographics • Dates and results of screening and diagnostic tests • Final diagnosis
What strategies are grantees implementing?	Grantee survey	Annually	Grantee	• Grantee characteristics • Implementation of evidence-based interventions • Partnerships
Are state-level colorectal cancer screening rates increasing?	Behavioral Risk Factor Surveillance System	Every 2 years	State	CRC screening rate
What is the cost of delivering the CRCCP?	Cost assessment tool	Annually	Grantee	• Costs of screening provision • Costs of screening promotion

## CRCCP Evaluation Findings

In this collection, 5 articles address aspects of 4 evaluation questions: 1) Is complete and timely screening delivered, and what are the screening outcomes? 2) What strategies are grantees implementing? 3) Are state-level colorectal cancer screening rates increasing? and 4) What is the cost of delivering the CRCCP? Nadal et al assessed the quality of screening services provided through the screening provision component of the program (17). On the basis of accepted standard practices, they analyzed CCDE data collected by CDC on the timing and results of all screening and diagnostic tests provided and the quality of colonoscopies provided. Researchers found that most positive results for FOBTs and FITs were appropriately followed up with colonoscopy to complete the screening process, and most of the colonoscopies were completed within the time frame of 180 days recommended by CDC. Additionally, the authors found that most colonoscopies performed met national quality standards. Although most quality indicators were met by grantees, quality varied substantially across grantees. The article discusses the challenges of modifying the behaviors of health care providers to improve the quality of services provided.

Hannon et al analyzed data from grantee surveys to examine use of EBIs and facilitators and barriers to implementation (18). The authors found that most grantees implemented and maintained client-oriented EBIs such as client reminders and small media. Grantees considered these EBIs easier to implement than provider-oriented EBIs or reduction of structural barriers. Unexpectedly, implementation of EBIs did not become easier over time, possibly because of the need to build and sustain partnerships over time with health care providers and organizations.

Three articles evaluated the cost of delivering the CRCCP. Hoover et al described the development of a web-based cost-assessment tool to collect cost data and evaluate the quality of the data collected by the tool (19). The authors found that most grantees were able to use the tool to allocate at least 95% of the funds they received to program activities. Keys to successful implementation of the tool were solicitation of grantee input during the development and design phases and staff members dedicated to providing technical assistance to grantees. Subramanian et al described the clinical and nonclinical costs of the direct screening services provided (ie, screening provision) by grantees (20). Although the authors found that direct clinical costs were higher for colonoscopy-only screening programs than for FOBT/FIT-only programs, nonclinical costs did not vary by screening test type, suggesting that these programs have substantial fixed costs. Finally, Tangka et al examined differences in grantees’ expenditures for screening promotion (21). Researchers found that grantees allocated nearly one-third of their funding to screening promotion activities that had insufficient evidence of effectiveness (eg, mass media) as determined by *The Community Guide* (14) and smaller amounts were allocated toward recommended interventions (eg, small media, provider assessment and feedback, client and provider reminders).

The 2009–2015 CRCCP was the first public health program focused solely on increasing use of CRC screening tests at the population level in multiple states by supporting both direct CRC screening services and CRC promotion through implementation of EBIs. The findings from the articles in this collection provide important information that can inform future programs of the type and scope of the CRCCP. First, although grantees were successful in providing high-quality screening services directly to more than 50,000 people who had limited or no health insurance, the cost of program infrastructure was high, and the number of people screened was much lower than the number of people who were eligible for the program. This finding led CDC to decrease funding for direct screening services in the current CRCCP (2015–2020) and focus on implementation of EBIs in primary care clinics to reduce program infrastructure costs while potentially increasing program reach. Second, we found that most programs did not have state-wide reach and most were unable to measure changes in uptake of CRC screening tests by using a population measure such as the Behavioral Risk Factor Surveillance System. As a result, the 2015–2020 CRCCP requires grantees to partner directly with health systems and primary care clinics that serve populations known to have low CRC screening test use (eg, federally qualified health centers) to implement EBIs and to report clinic-level CRC screening data to measure success. This change also allows grantees to narrow the scope of their programs by focusing on high-need populations while still potentially expanding their overall reach. Third, we found that grantees allocated a disproportionate amount of their awards toward interventions with limited evidence for their effectiveness (eg, mass media), and grantees found client-oriented interventions, such as client reminders and small media, easier to implement. The 2015–2020 CRCCP now requires that grantees choose at least 2 of 4 priority EBIs (client reminders, provider assessment and feedback, provider reminders, and reduction of structural barriers) that have sufficient or strong evidence of effectiveness in increasing CRC screening. The 2015–2020 CRCCP grantees are strongly encouraged to partner with various organizations, such as primary care associations, ACS, and entities with expertise in health information technology, to facilitate the implementation of both client-oriented and provider-oriented EBIs in primary care clinics.

The evaluation findings from the 2009–2015 CRCCP were critical to inform the design and implementation of the 2015–2020 CRCCP (22). The usefulness of the findings demonstrates the importance of a well-designed and executed evaluation plan. Although the 2009–2015 CRCCP was unique in its design, size, and scope, these evaluation findings can be useful to other public health organizations planning or implementing similar population-level interventions to increase CRC screening. Program planners should carefully consider the potential reach and infrastructure costs of direct CRC screening services given available sources of funding, the size of the potential target population relative to the capacity and funding of program implementers, the selection of EBIs that maximize program effects while minimizing costs, and the ability of program implementers to leverage the resources of other public and nonpublic health organizations to facilitate implementation. Evaluation should be an integral part of program planning and should answer questions about how the program was implemented and its effectiveness. Evaluation findings from programs such as the CRCCP are vital to demonstrate the effectiveness of public health programs in addressing the burden of CRC in the United States.
